# Fatal Poisoning by Inhalation of Carbolic Acid

**Published:** 1881-12

**Authors:** 


### FATAL POISONING BY INHALATION OF CARBOLIC ACID.

Dr. F. P. Blake, of Canon City, Colorado, reports, in the *Rocky Mountain Medical Review*, for July, 1881, the case of a lady, aged 29½ years, married and near the full term of pregnancy, who was found dead in bed. A handkerchief enclosing a sponge was in close proximity to her face, which was cyanosed. The tip of the nose was blistered from contact with the substance contained in the inhaler. And odor of carbolic acid prevaded the room ; the sponge and handkerchief were saturated with the odor, though dry when found. No one was in the room when the woman died. She was subject to neuralgia, and it is supposed that she sought relief by inhaling the acid, which asphyxiated her.

The appearance of the mouth did not indicate that the acid had been taken internally.

